# The Rise in Norovirus-Related Acute Gastroenteritis During the Fight Against the COVID-19 Pandemic in Southern China

**DOI:** 10.3389/fpubh.2021.785373

**Published:** 2022-01-11

**Authors:** Ying Lu, Zhoubin Zhang, Huaping Xie, Wenzhe Su, Hui Wang, Dahu Wang, Jianyun Lu

**Affiliations:** ^1^Department for Infectious Diseases Control and Prevention, Guangzhou Center for Disease Control and Prevention, Guangzhou, China; ^2^Director's Office, Guangzhou Center for Disease Control and Prevention, Guangzhou, China; ^3^Department of Virology and Immunology, Guangzhou Center for Disease Control and Prevention, Guangzhou, China

**Keywords:** outbreaks, clusters, norovirus, acute gastroenteritis, COVID-19

## Abstract

**Background:** There has been a significant decline in the morbidity of almost all infectious diseases during the COVID-19 pandemic. However, while the incidence of norovirus-related acute gastroenteritis declined in Guangzhou, China during the initial period of the pandemic, incidence increased significantly once the new school year began in September 2020.

**Methods:** Norovirus-related acute gastroenteritis clusters and outbreaks were assessed in Guangzhou from 2015 to 2020. Medians and interquartile ranges were compared between groups using the Mann–Whitney *U*-test, and attack rates were calculated.

**Results:** While 78,579 cases of infectious diarrhea were reported from 2015 to 2019, with an average of 15,716 cases per year, only 12,065 cases of infectious diarrhea were reported in 2020. The numbers of sporadic cases and outbreaks reported from January to August 2020 were lower than the average numbers reported during the same time period each year from 2015 to 2019 but began to increase in September 2020. The number of cases in each reported cluster ranged from 10 to 70 in 2020, with a total of 1,280 cases and an average attack rate of 5.85%. The median number of reported cases, the cumulative number of cases, and the attack rate were higher than the average number reported each year from 2015 to 2019. The intervention time in 2020 was also higher than the average intervention time reported during 2015–2019. The main norovirus genotypes circulating in Guangzhou during 2015–2020 included genogroup 2 type 2 (GII.2) (*n* = 79, 26.69%), GII.17 (*n* = 36, 12.16%), GII.3 (*n* = 27, 9.12%), GII.6 (*n* = 8, 2.7%), GII.4 Sydney_2012 (*n* = 7, 2.36%), and GII.4 (*n* = 6, 2.03%).

**Conclusions:** Our findings illustrate the importance of maintaining epidemiological surveillance for viral gastroenteritis during the COVID-19 pandemic. Local disease prevention and control institutions need to devote sufficient human resources to control norovirus clusters.

## Background

The novel COVID-19 epidemic began in Wuhan city, China in December 2019 and spread rapidly throughout the world. Human safety and health faced a major threat. A series of public social distancing interventions were implemented (1) that may have implications for the management of non-COVID-19 diseases.

Norovirus, also called the “winter vomiting bug,” is the leading cause of non-bacterial gastroenteritis outbreaks worldwide (2), affecting individuals of all ages. Noroviruses are responsible for a considerable burden of viral gastroenteritis in China, especially in people < 2 and over ≥ 65 years of age (3). Noroviruses are highly contagious with virus shedding sometimes lasting several weeks, however, the course of infection is self-limited, and patients usually recover within 2–3 days (4). Transmission occurs through the fecal-oral route, either by direct contact with infected individuals, or contaminated surfaces, food, water, and aerosols (4, 5). The majority of reported outbreaks in China from 2014 to 2017 occurred in school settings including primary schools, childcare facilities, and secondary schools (6).

During the COVID-19 pandemic, there was a major decline in the morbidity of almost all respiratory infectious diseases, including varicella, rubella, measles (7), and influenza (8), likely because the route of transmission is similar to COVID-19. A total of 39 notifiable infectious diseases were reported in Guangdong during the COVID-19 emergency response period in 2020, a 50.7% reduction from the same period in 2015–2019 (9). However, the incidence of norovirus-related acute gastroenteritis only declined during the initial period. When the school opened in September, the number of norovirus clusters increased significantly in Guangzhou, southern China.

In this study, the annual incidence of infectious diarrhea in Guangzhou was compared between the period before the COVID-19 pandemic, 2015 to 2019, and the period after, 2020, to assess whether additional strategies are required to prevent and control the spread of infectious diarrhea.

## Methods

### Data Collection

Infectious diseases causing diarrhea, including norovirus, are regarded as class C notifiable infectious diseases in China. Suspected and confirmed cases identified by medical institutions must be reported to the National Notifiable Infectious Disease Reporting Information System (NIDRIS) within 24 h. According to the Norovirus Outbreak Management and Disease Prevention Guidelines (2015 version) (10) compiled by the Chinese Center for Disease Control and Prevention (China CDC) and Guidelines for prevention and control of norovirus infectious diarrhea in Guangdong Province (2015 version) (11), norovirus clusters are defined as then or more cases of norovirus infectious diarrhea (including clinical or laboratory-confirmed) occurring in the same school, kindergarten, nursing home, natural village, community, factory, construction site, or other collective units within 1 week. Norovirus outbreaks are defined as 20 or more cases of norovirus infectious diarrhea (including clinical or laboratory-confirmed) occurring in the same school, kindergarten, nursing home, natural village, community, factory, construction site, and other collective units within 1 week. On September 8, 2020, the definition was adjusted to 10 or more cases of infectious diarrhea or vomiting occurring in the same school, kindergarten, natural village, community, construction site, or other collective units within 3 days in order to improve the sensitivity of infectious disease reporting. Data on reported cases of infectious diarrhea was obtained from NIDRIS, and information on norovirus clusters was obtained through ongoing daily surveillance.

### Case Definition

According to the Diagnostic Criteria for infectious diarrhea (WS271-2007) from the Chinese Ministry of Health, infectious diarrhea is a group of intestinal infectious diseases caused by pathogenic microorganisms, including bacteria, viruses, and parasites, and is often characterized by diarrhea, and it only refers to infectious diarrhea except for cholera, dysentery, typhoid, and paratyphoid.

### Data Analysis

The epidemic scale was defined as the cumulative number of reported cases. Intervention time was defined as the time from the first case's disease onset to the time when intervention was initiated. The epidemic duration was defined as the time from the first to the last case's disease onset. Schools start in different batches beginning in May 2020, so the incidence of infectious diarrhea occurring from January to April 2020 was compared with the mean incidence occurring during the same time periods in 2015–2019. Chi-square statistical tests were used to compare rates. Median and interquartile ranges were compared between groups using the Mann–Whitney *U*-test. Attack rates referred to the disease incidence occurring in a short time period in a limited area by dividing the number of new cases in the observation period by the number of exposed individuals in the same period (12). All statistical tests were two-sided, and *P* < 0.05 were considered statistically significant. All analyses were performed using R 3.2.1 (The R Project for Statistical Computing, Vienna, Austria).

## Results

### Comparative Analysis of Infectious Diarrhea Occurring in 2020 With the Prior 5 Years

**Figure 1:**
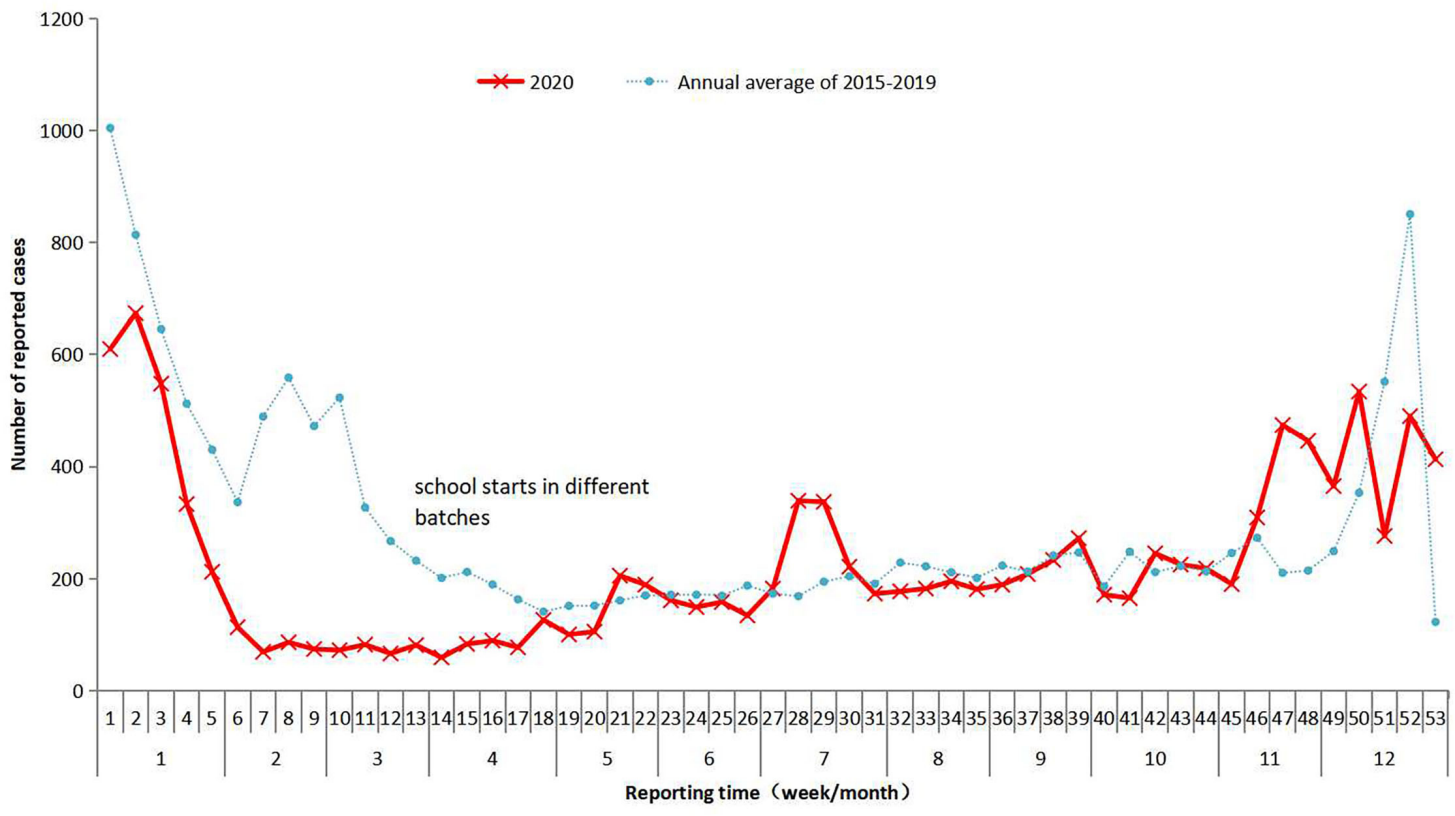
The monthly number of reported cases of infectious diarrhea in Guangzhou, 2015–2020. The average annual number of reported cases of infectious diarrhea in Guangzhou during 2015–2019 is represented by the blue dotted line. The monthly number of reported cases of infectious diarrhea in Guangzhou in 2020 is represented by the red line.

**Figure 2:**
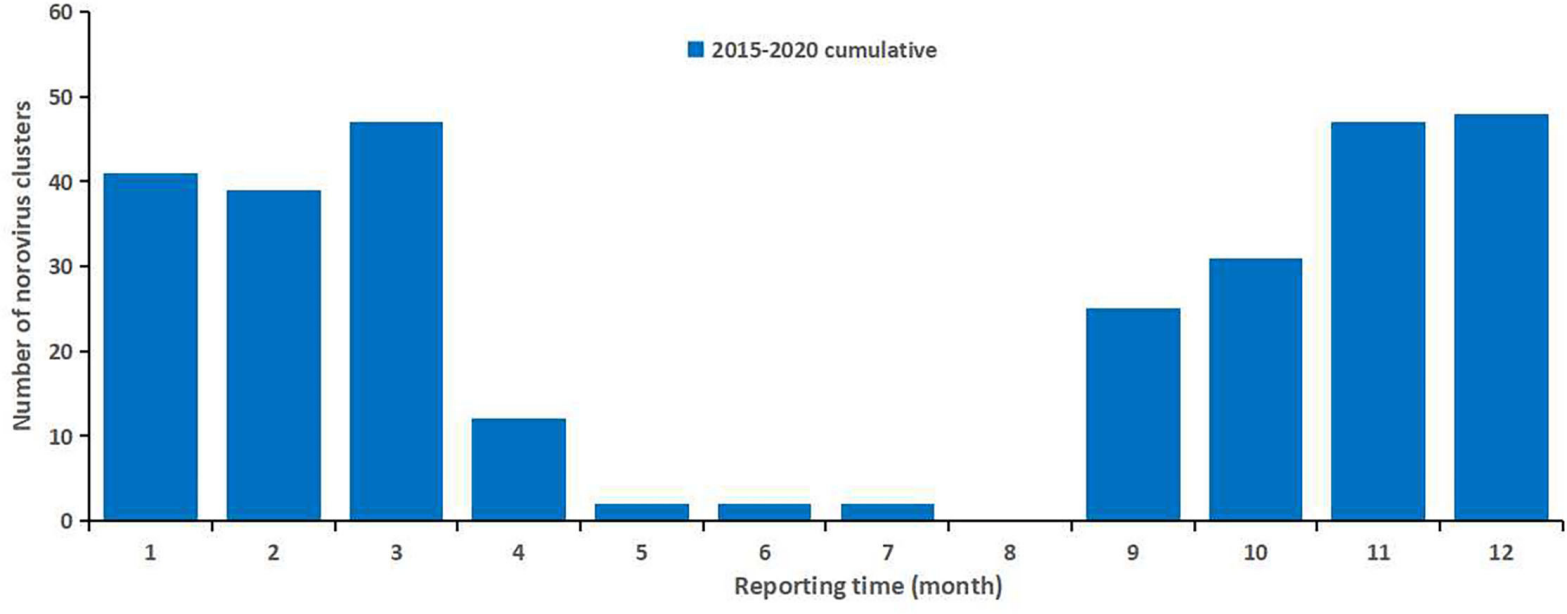
The monthly number of norovirus clusters reported in Guangzhou, 2015–2020. The height of the blue column represents the number of clusters. The peak occurred from November to March of the subsequent year.

While 78,579 cases of infectious diarrhea were reported to NIDRIS from 2015 to 2019, with an average of 15,716 cases per year, only 12,065 cases of infectious diarrhea were reported in 2020. From January to August 2020, the number of reported cases was lower than the average number reported during the same time periods in 2015–2019, however, after September 2020, the number of reported cases gradually increased above the average level seen during the same time periods in 2015–2019 (Figure 1). Clustered outbreaks reported in 2020 followed a similar trend, with a lower-than-average number reported during January to August 2020 than was reported during the same time periods from 2015 to 2019, and a higher number reported after September (Figure 2). The incidence of infectious diarrhea reported from January to April 2020 was significantly lower than the incidence reported during the same periods from 2015 to 2019 (χ^2^ = 2180.128, *P* < 0.01).

### Epidemiological Characteristics of Clusters

In 2020, the number of reported cases associated with each cluster ranged from 10 to 70 (median 18 cases), with a total of 1,280 cases and an average attack rate of 5.85%. The average annual median number of 17 reported cases per cluster, 670 total cases, and 4.83% attack rate were all higher during 2015–2019. The intervention time for clustered cases ranged from 0 to 11 days, with a median of 3 days, which was higher than the average intervention time of 3 days reported during 2015–2019, but not statistically significant (*P* = 0.051). Only 11 (20%) of the clusters received intervention within 1 day, 26 (47.3%) within 2–3 days, and 18 (32.7%) after 3 days. The median duration of the clusters was 13 days, which was significantly higher than the 9 days observed from 2015 to 2019. Among these, two clusters (3.7%) lasted <7 days, 17 clusters (31.5%) lasted 7–10 days, and 64.8% lasted more than 10 days (Table 1).

**Table 1:** Epidemiological characteristics of clusters from 2015 to 2020.

**Variables**	**2015**	**2016**	**2017**	**2018**	**2019**	**2020**	**2015–2019**	**P**
**Epidemic scale**								
Median (P25–P75)	15	15.5	16	15	17	18 (14–25)	15 (12–19)	0.005
Annual average cumulative cases	1788	855	1302	995	670	1280	1122	
Annual average attack rate	3.28%	5.10%	6.13%	5.07%	4.83%	5.85%	4.88%	
**Intervention time**								
Median (P25–P75)	/	/	2	2	3	3 (2–5)	2 (1–3)	0.051
≤ 1 day	/	/	24 (42.9%)	14 (31.8%)	8 (24.2%)	11 (20.0%)	46 (34.6%)	0.126
2–3 days	/	/	21 (37.5%)	21 (47.7%)	13 (39.4%)	26 (47.3%)	55 (41.4%)	
>3 days	/	/	11 (19.6%)	9 (20.5%)	12 (36.4%)	18 (32.7%)	32 (24.1%)	
**The duration of the epidemic**								
Median (P25–P75)	9	/	8	7	10	13(9.75–17.25)	9(6–13)	<0.01
≤ 6 days	17 (23.6%)	/	18 (32.7%)	17 (41.5%)	4 (12.1%)	2 (3.7%)	56 (27.9%)	<0.01
7–10 days	26 (36.1%)	/	14 (25.5%)	13 (31.7%)	16 (48.5%)	17 (31.5%)	69 (34.3%)	
>10 days	29 (40.3%)	/	23 (41.8%)	11 (26.8%)	13 (39.4%)	35 (64.8%)	76 (37.8%)	

*Medians were compared using Mann-Whitney U test between the two groups of 2020 and 2015-2019. Chi-square statistical tests were used to compare the different distribution of intervention time and the duration of the epidemic. Data of the duration of the epidemic for 2016 are unavailable and missing. The p value of 0.126 is the comparison between the three groups of intervention time, and the p value of <0.01 is the comparison between the three groups of the duration of the epidemic*.

### Molecular Characterization of Norovirus Circulating in Guangzhou, China, During 2015-2020

**Figure 3:**
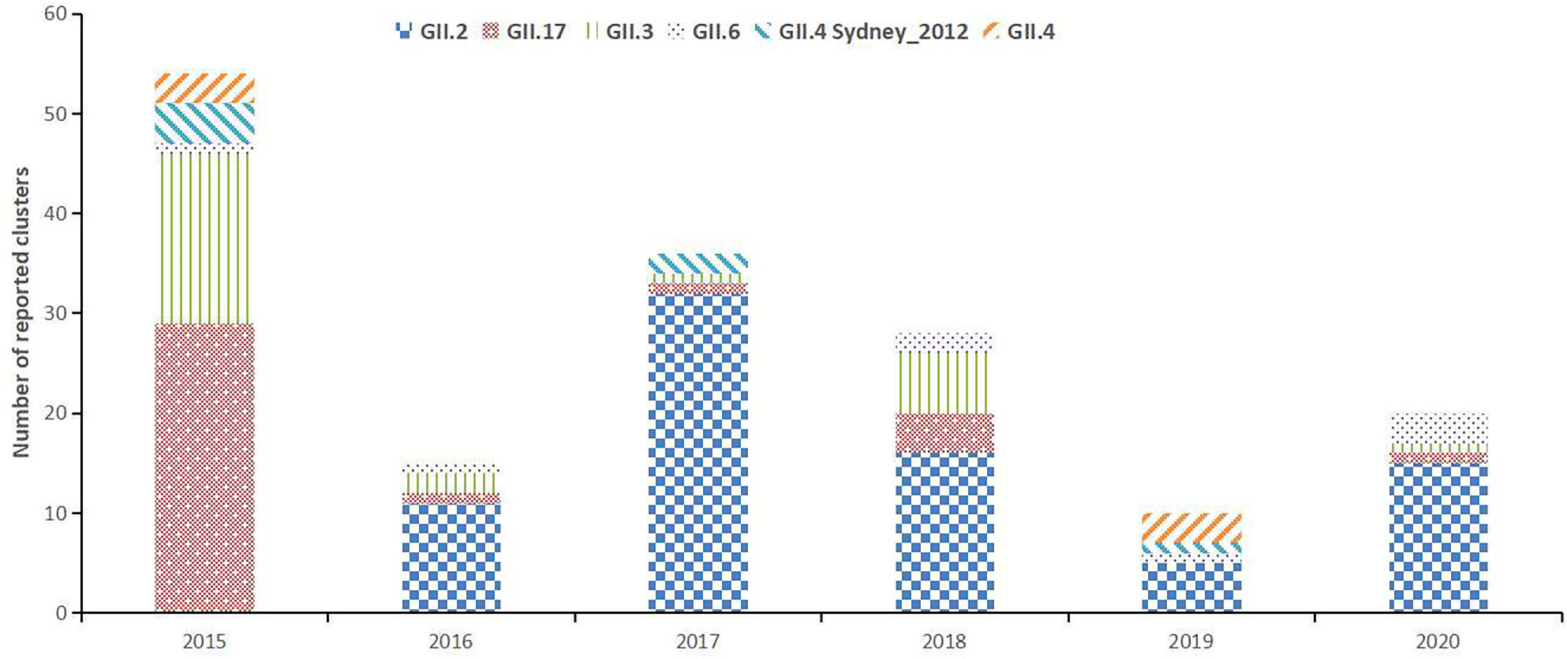
The annual distribution of the molecular genotypes of norovirus clusters in Guangzhou, China, 2015–2020. Columns show the number of cases infected with each genotype.

Among the 296 norovirus-related acute gastroenteritis clusters, 287 (96.96%) were norovirus genogroup 2 (GII) and three (1.01%) were norovirus GI. Four cases were infected with both norovirus GI and GII, and two cases were infected with both GII and sapovirus. The main norovirus genotypes circulating in Guangzhou during 2015–2020 included GII.2 (*n* = 79, 26.69%), GII.17 (*n* = 36, 12.16%), GII.3 (*n* = 27, 9.12%), GII.6 (*n* = 8, 2.7%), GII.4 Sydney_2012 (*n* = 7, 2.36%), and GII.4 (*n* = 6, 2.03%). Other identified strains were GI.2, GII.7, and GII.14 (Figure 3).

## Discussion

Norovirus activity, including both outbreaks and sporadic norovirus infections, decreased in 2020, particularly at the beginning of the COVID-19 pandemic. Eigner et al. (13) reported that the reduction in norovirus infections reported in Germany in 2020 followed the implementation of COVID-19 containment measures. Douglas et al. (14) also reported a substantial and sustained reduction in norovirus outbreaks during 2020 after severe acute respiratory syndrome coronavirus-2 (SARS-CoV-2) control measures were first introduced in England. The incidence of enteroviruses and norovirus, which are transmitted primarily through the fecal-oral route, declined significantly in Victoria, Australia, in 2020 as compared to the previous decade (15). Similarly, there was a reduction in sporadic norovirus infections after the COVID-19 pandemic started in the United States (16).

Prior studies only reported on norovirus activity during the early stage of the COVID-19 pandemic. Once prevention and control became normalized, norovirus characteristics were no longer reported. The current study describes norovirus-related acute gastroenteritis surveillance data collected during the 6-year period from 2015 to 2020 in Guangzhou, southern China. The number of reported cases of infectious diarrhea reported at the start of 2020 was significantly lower than the average number reported during the same time periods in the prior 5 years. It is possible that health care systems strained by the COVID-19 pandemic indirectly impacted access to health care, including visits to emergency departments, for patients with other conditions (8, 17). China enacted strict lockdown policies to prevent and slow down person-to-person transmission in order to control the pandemic. This resulted in a decline in overall healthcare, especially during the post-festival period in 2020, that gradually increased once lockdown policies were loosened (18).

While the numbers of norovirus cases and clusters reported from January to August 2020 were significantly lower than the numbers reported during the equivalent time periods in 2015 to 2019, the numbers of reported cases and clusters reported after schools reopened in September 2020 were significantly higher than the numbers reported during equivalent time periods in the past 5 years. China implemented intense non-pharmaceutical interventions to stop COVID-19 transmissions, such as social distancing and school closure, which changed patterns of human contact (1). These measures had a positive effect on the control of norovirus-related acute gastroenteritis since outbreaks predominantly occur in schools, childcare centers, healthcare facilities, and other crowded settings (10, 19). The risk of infectious diarrhea transmission rises in children when they return for the new school year. Norovirus is primarily transmitted through the fecal-oral route and patients usually shed norovirus by contaminating food and surfaces. Thus, hand washing, thorough cooking, and environmental cleaning and disinfection are effective means to prevent and control disease spread.

While control measures such as social distancing and mask mandates have lowered the risk of some respiratory infectious diseases, these precautions have a limited effect on the prevention and control of intestinal infectious diseases. In addition, many regions in China have introduced policies one after another, that is primary and secondary school students were no longer required to wear masks on campus once prevention and control were normalized. It is also possible that during COVID-19, some schools focused less attention on gastrointestinal infectious disease control measures. As a result, schools may have failed to find students and staff experiencing gastrointestinal discomfort and report potential cases before they were able to transmit disease and initiate outbreaks. These findings highlight the importance of maintaining robust viral gastroenteritis surveillance during the COVID-19 pandemic and remaining alert to the emergence of novel norovirus strains once control measures were relaxed.

In this study, the median number of reported cases, the cumulative number of cases, and the attack rate were higher during the final months of 2020 than during the same periods each year from 2015 to 2019, indicating that the average scale of clusters during the epidemic showed an upward trend. In addition, there were fewer clusters with an intervention time < 1 day in 2020 than in 2019, suggesting that COVID-19 prevention and control may have occupied human and material resources needed for response to other infectious diseases, such as infectious diarrhea. The large basic reproduction number of noroviruses facilitates the initiation of large outbreaks in childcare institutions and schools (20). Thus, it is critical to take appropriate prevention and control measures, including case management, hand hygiene, environmental disinfection, food and water safety, risk assessment, and health education, during the early stages of an outbreak.

Human norovirus is classified into three genogroups, GI, GII, and GIV. GII type 4 (GII.4) is the most prevalent worldwide (21) and is the genogroup reported in 62.8% of total outbreaks. In the United States, the main non-GII.4 genotypes that occurred from 2009 to 2016 were GI, GII.6, and GII.2 (22). A report from Japan showed that norovirus GII.6 emerged as the second most common strain after GII.4, 2008–2009 (23). The common genotypes that occurred in 73 norovirus outbreaks in Guangdong province, China, 2008–2015 were GII.4/Sydney 2012, GII.17, and GII.3 (24). The predominant norovirus genotypes changed each year and the main non-GII.4 genotypes co-circulated with GII.4 in Huzhou China, 2016–2017 (25). New variants often increase the number of norovirus outbreaks and result in more severe health outcomes (26). While many infectious diseases declined substantially owing to COVID-19-specific control measures (9), the increased norovirus activity observed after control measures were relaxed in Guangzhou requires attention. Ongoing surveillance of circulating norovirus strains should be strengthened.

District-level disease control and prevention institutions and community health service centers require sufficient human resources for norovirus control on the premise of completing the normalized prevention and control of COVID-19. This will help to shorten norovirus outbreak response times in order to control transmission more quickly and effectively.

## Conclusions

The importance of maintaining surveillance for viral gastroenteritis during the COVID-19 pandemic should be emphasized. Local disease prevention and control institutions need to dedicate sufficient human resources to prevent potential outbreaks.

## Data Availability Statement

The raw data supporting the conclusions of this article will be made available by the authors, without undue reservation.

## Author Contributions

YL, ZZ, and JL: concept and design. YL, DW, and HW: acquisition, analysis, or interpretation of data. YL and DW: drafting of the manuscript. HX and WS: experiment. YL and HW: statistical analysis. JL: supervision. All authors have read and approved the final manuscript.

## Funding

This study was supported by the Key Project of Medicine Discipline of Guangzhou (No. 2021-2023-11), Medical Health Technology Project for Guangzhou (No. 20201A011062), and Medical Science and Technology Foundation of Guangdong Province (No. A2021372).

## Conflict of Interest

The authors declare that the research was conducted in the absence of any commercial or financial relationships that could be construed as a potential conflict of interest.

## Publisher's Note

All claims expressed in this article are solely those of the authors and do not necessarily represent those of their affiliated organizations, or those of the publisher, the editors and the reviewers. Any product that may be evaluated in this article, or claim that may be made by its manufacturer, is not guaranteed or endorsed by the publisher.
